# Identification of Genetic Relationships and Group Structure Analysis of Yanqi Horses

**DOI:** 10.3390/genes16030294

**Published:** 2025-02-27

**Authors:** Yaru Wang, Chi Tang, Pengfei Xue, Na Yang, Xiaoyuan Sun, Khizat Serik, Tolegen Assanbayer, Malika Shamekova, Zhassulan Kozhanov, Zagipa Sapakhova, Jurakulov Kobil Khurramovich, Xiaoling Zhou, Iskhan Kairat, Gemingguli Muhatai

**Affiliations:** 1 College of Animals Science and Technology, Tarim University, Alar 843300, China; wyr110418@163.com (Y.W.);; 2 Key Laboratory of Tarim Basin Biological Resources Protection and Utilization, Tarim University, Alar 843300, China; 3 Key Laboratory of Tarim Livestock Science and Technology Corps, Tarim University, Alar 843300, China; 4 Physiology, Morphology and Biochemistry, Kazakh National Agrarian Research University, Almaty 050010, Kazakhstan; 5 Zootechnology and Veterinary Medicine, Toraighyrov University, Pavlodar 140008, Kazakhstan; 6 Institute of Plant Biology and Biotechnology, Breeding and Biotechnology Laboratory, Almaty 050000, Kazakhstan; 7 Horse Breeding Department, Kazakh Research Institute of Livestock and Forage Production, Almaty 050000, Kazakhstan; 8 Animal Husbandry and Biotechnology, Samarkand State University of Veterinary Medicine, Samarkand 140100, Uzbekistan

**Keywords:** Yanqi horse, microsatellite markers, paternity testing, genetic diversity, genetic structure

## Abstract

**Background/Objectives:** The Yanqi horse is a distinguished local breed in China, known for its robust physique and strong adaptability. However, due to insufficient breeding populations and a loosely structured breeding system, the number of Yanqi horses has been declining annually. To protect its genetic resources and develop scientific breeding strategies, this study aimed to analyze the genetic diversity, parentage relationships, and genetic structure of the Yanqi horse conservation population using microsatellite markers. **Materials and Methods:** A total of 117 Yanqi horses were selected for genotyping analysis using 16 microsatellite markers. Genetic diversity parameters (e.g., allele number, heterozygosity, F-statistics) were calculated using GeneAIEX (v.6.503) and Fstat software (v.2.9.4). Parentage analysis was conducted using Cervus software. Bayesian clustering analysis was performed using STRUCTURE software (v.2.3.4), and a phylogenetic tree was constructed based on Nei’s genetic distance to reveal the population genetic structure. **Results:** A total of 191 alleles were detected, with an average allele number of 11.969, observed heterozygosity of 0.481, and expected heterozygosity of 0.787. Parentage testing showed a cumulative exclusion probability (CEP) of 0.9652999 when one parent’s genotype was known and 0.9996999 when both parents’ genotypes were known, achieving an accuracy of 99%. Genetic differentiation analysis revealed moderate genetic divergence among populations (FST = 0.128) and moderate inbreeding levels (FIS = 0.396). Bayesian clustering analysis (K = 4) indicated that the Yanqi horse population could be divided into four genetic clusters, reflecting the impact of geographical isolation on genetic structure. **Conclusions:** The Yanqi horse conservation population exhibits moderate genetic diversity, high accuracy in parentage identification, and moderate genetic differentiation and inbreeding. The findings provide a scientific basis for the conservation and sustainable utilization of Yanqi horse genetic resources. Future efforts should focus on strengthening conservation measures, optimizing breeding strategies, and further investigating the genetic background using genomic technologies to ensure the sustainable development of the Yanqi horse population.

## 1. Introduction

Horses (*Equus caballus*) serve as a significant catalyst for cultural exchanges and the advancement of human society. Concurrently, human activities have profoundly influenced the population structure and evolutionary dynamics of horse groups. As human demand for horses evolves, breeding strategies are adapted accordingly, leading to the differentiation of genetic structures among various breeds and the emergence of breeds with distinct phenotypes [1]. Currently, due to the indiscriminate introduction of horses for commercial purposes, local horse breeds face critical challenges, including the loss of genotypes and severe breed assimilation. According to the 2022 statistics from the Food and Agriculture Organization of the United Nations (FAO), over 88 local horse breeds have become extinct. Therefore, it is crucial to preserve the genetic characteristics and protect the genetic diversity of valuable local horse breeds, such as the Yanqi horse, through scientific and technical interventions.

China is among the countries with the richest horse resources globally, benefiting from a favorable geographical location, a robust natural ecological environment, and diverse topography, landforms, and climates. As a result, various horse breeds have emerged, including grassland types (such as Kazakh and Mongolian horses), mountain types (such as Jianchang and Ningqiang horses), and forest types (such as the Oroqen horse) [2]. However, with the rapid advancement of agricultural mechanization and modernization, mechanical farming and transportation have increasingly supplanted animal power. This shift has led to a significant reduction in the number of military and agricultural draft horses, contributing to a decline in the horse population within the breeding industry, with some local breeds facing the threat of extinction. To address this issue and protect local horse breeds with exceptional traits, seven breeds have been designated for inclusion in the national livestock and poultry genetic resources protection list, with key protective measures implemented (See List S1 for specific listings).

**Figure 1:**
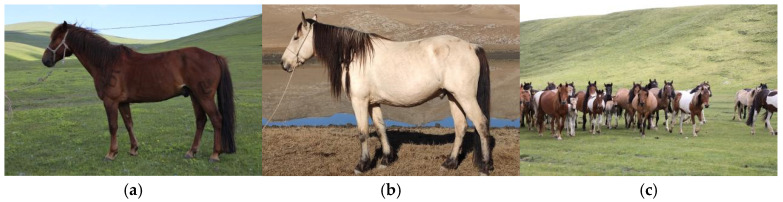
(**a**) Male Yanqi horse; (**b**) mare Yanqi; and (**c**) a group photo of Yanqi horses.

The Yanqi horse is native to the hinterland of the Tianshan Mountains, located in the northern part of the Bayingolin Mongolian Autonomous Prefecture in the Xinjiang Uygur Autonomous Region of China. This breed boasts a long history and unique genetic characteristics. The development of the Yanqi horse is closely linked to the migration history of the Mongolian people, with its bloodline incorporating genes from various horse breeds, including Mongolian, Kazakh, and Circassian horses [3]. The Yanqi horse is renowned for its exceptional traits, such as strong adaptability, resistance to rough feeding, and a high reproductive rate. The morphological characteristics of Yanqi horses are shown in Figure 1. It features a compact body structure, a well-proportioned physique, erect ears, and an agile yet docile temperament, making it highly valued by horse breeders. Furthermore, the Yanqi horse serves not only as a vital tool for local agricultural production but also as an integral component of Xinjiang’s horse culture. Therefore, its protection and research are of significant importance for maintaining biodiversity and promoting local economic development.

The Yanqi horse is a local fine breed that has developed through long-term selection and breeding, shaped by the region’s natural conditions as well as its horse culture and national history. Li Hangsen (2021) conducted a study on the phenotypic diversity of Yanqi horses, classifying them into two types: mountain type and plain type. He discovered that mountain-type Yanqi horses exhibit a rich diversity of coat colors and can be bred for endurance, leisure riding, and both meat and milk production [4]. Cairendaoerji and Guan Yongping (2014) examined the endurance and other germplasm characteristics of the Yanqi horse, proposing a breeding plan and highlighting the urgency of protecting the genetic resources of this breed [5,6]. The 70K SNP chip was utilized to study nine domestic and foreign horse breeds, including the Yanqi horse. This research revealed that the Yanqi horse displays the highest degree of differentiation from purebred horses and shares the closest genetic structure with the Yili horse. Additionally, it was found that the Yanqi horse population is characterized by a low level of inbreeding. However, the number of effective populations is declining rapidly, necessitating immediate conservation measures to prevent the loss of genetic diversity and the onset of inbreeding depression [7]. Current research on Yanqi horses primarily focuses on disease prevention and treatment [8,9,10], breed selection [11], gait genes [12,13], and phenotypic studies [14]. Thus, to preserve its genetic uniqueness and mitigate further loss of genetic diversity, additional genetic research is essential to inform the development of relevant conservation and breeding programs.

Microsatellite DNA markers, characterized by their high degree of polymorphism, low cost, and straightforward operational procedures, play a crucial role in the study of horse population structure and genetic diversity. This technique has been extensively employed in equine genetics research since 1994 [9]. In recent years, while some studies have demonstrated that SNPs and gene chips can be utilized for paternity identification, there remain certain limitations regarding exclusion probabilities at specific loci [15,16]. Consequently, microsatellite markers continue to serve as a vital tool for equine genetic identification and are recognized globally as the most commonly used and accurate method for paternity testing [17,18]. In this study, microsatellite marker technology was employed to analyze the genetic relationships of Yanqi horses, aiming to elucidate their genetic diversity and population structure and to provide a scientific theoretical foundation and practical guidance for the breeding and enhancement of Yanqi horses. This research offers more precise scientific support for the conservation, development, and utilization of genetic resources in other local horse breeds.

## 2. Materials and Methods

Sample collection and measurement: A total of 117 Yanqi horses (Picture c), comprising 40 males (Picture a) and 76 females (Picture b), were randomly selected from Xinjiang, China. There are 57 horses at the Baozhou Baoqi Yanqi Horse Conservation Farm in Bazhou, 41 horses in Hejing County, 14 horses in Heshuo County, and 9 horses in Bohu County. All selected horses exhibited good body condition and were subjected to similar feeding and management practices. Blood samples (5 mL) were collected from the jugular vein by a licensed veterinarian using EDTA anticoagulant tubes, which were then transferred to cryopreservation tubes and stored at −80 °C for future analysis.

DNA extraction from the collected blood samples was performed using a DNA extraction kit (Tiangen Biochemical Technology (Beijing, China) Co., Ltd.: DP304 kit). An Eppendorf biospectrophotometer (Hamburg, Germany) was utilized to quantify the extracted DNA samples, which were then stored at −20 °C for subsequent analysis.

The International Society for Animal Genetics and the FAO International selected 16 microsatellite loci with two-base repeats (*HMS7*, *HTG10*, *ABS23*, *HMS3*, *AHT4*, *HMS2*, *TKY297*, *ASB17*, *AHT5*, *ABS2*, *HMS9*, *VHL20*, *HMS6*, *HMS18*, *TKY343*, *TKY337*) in Table S1 for PCR amplification. In this experiment, the primer design software Primer Premier V5.0 [19] was utilized, and some primers were redesigned. All primers were synthesized by Shanghai Sangon Biotechnology Services Co., Ltd. (Shanghai, China). A grouped multiplex PCR amplification reaction was performed. The PCR reaction system consisted of 25 µL, which included 2 µL of DNA template, 1 µL each of upstream and downstream primers, 12.5 µL of Supermix PCR buffer, and 9.5 µL of ddH2O. The reaction process involved an initial denaturation step at 95 °C for 5 min, followed by denaturation at 94 °C for 30 s. Each set of primers was then annealed at its optimal temperature for 30 s and extended at 72 °C for 30 s Specific information can be found in Table S2.

Data Analysis: The assessment of genetic diversity was conducted using the Gene AIEX software (version 6.5) [20]. A comprehensive suite of genetic diversity metrics were computed, encompassing allele frequency, the count of observed alleles (Na), the tally of effective alleles (Ne), the levels of observed heterozygosity (Ho), the estimates of expected heterozygosity (He), the Shannon information index (I), the fixation index (F), and the F statistics (FIS, FIT, FST) [21].

A phylogenetic tree was constructed using the Unweighted Pair Group Method with Arithmetic Mean (UPGMA) in MEGA5.05 software. The tree was subsequently visualized and refined on the ITOL (Interactive Tree Of Life) platform by adjusting branch colors, node styles, and label formats and adding annotation information (such as taxon names or evolutionary distances). Statistical and phylogenetic analyses were performed to explore the genetic structure and relationships within Yanqi horse family lines, utilizing the STRUCTURE software (version 2.3.4) [22,23]. To clarify the genetic architecture of Yanqi horses, the cluster matching and permutation program (clusterpp) [24] was applied to average the results from 15 replicates of each K value.

## 3. Result

### 3.1. Genetic Diversity Analysis

This study systematically evaluated the genetic diversity and kinship relationships of the Yanqi horse population based on 16 microsatellite loci. Genetic diversity analysis revealed a total of 191 alleles across all loci, with the number of alleles per locus (Na) ranging from 6 (*HMS18*) to 17.5 (*ABS17*) and the effective number of alleles (Ne) ranging from 2.959 (*HMS18*) to 12.040 (*ABS17*). Population-level genetic parameter analysis indicated an average number of alleles of 11.97 ± 1.11 and an average effective number of alleles of 7.27 ± 0.65, confirming that the selected loci have high polymorphic information content and are suitable for population genetic studies. Heterozygosity analysis showed that the average observed heterozygosity (Ho) was 0.48 (range: 0.29–0.83), while the average expected heterozygosity (He) was 0.79 ± 0.02 (range: 0.48–0.91). Notably, the He values were significantly higher than the Ho values across all loci (*p* < 0.05), a phenomenon that may be attributed to the Wahlund effect or inbreeding within the population. The data are summarized in Table 1 below. Further assessment of genetic diversity using the Shannon information index (I) revealed that the *VHL20* and *HMS18* loci exhibited the highest diversity (I = 0.86), while the *ABS23* locus showed the lowest diversity (I = 0.01). The average Shannon information index was 0.44, indicating moderate levels of genetic diversity within the population. Polymorphic information content (PIC) analysis further confirmed the suitability of the loci, with 14 loci (*HMS7*, *HTG10*, *ABS23*, *HMS3*, *AHT4*, *HMS2*, *TKY297*, *ABS17*, *AHT5*, *ABS2*, *HMS9*, *VHL20*, *HMS6*, *and TKY343*) classified as highly polymorphic (PIC > 0.5), and two loci (*HMS18* and *TKY337*) classified as moderately polymorphic (0.25 < PIC < 0.5). The differentiation coefficients (FSTs) of the loci ranged from 0.0016 to 0.0124, indicating strong discriminatory power within the population.

In summary, the 16 microsatellite loci used in this study exhibit the following characteristics: rich polymorphic information content, effectively reflecting population genetic diversity; moderate levels of differentiation among loci, suitable for population genetic structure analysis; and distinct heterozygosity distribution patterns, useful for detecting population genetic bottlenecks. These features demonstrate that the selected loci meet the technical requirements for parentage identification in Yanqi horses and provide a reliable molecular marker system for the conservation and management of this population’s genetic resources.

### 3.2. Kinship Analysis

The cumulative exclusion probability of 16 microsatellite loci was calculated using the Allele Frequency Analysis in Cervus 2.0 software. As shown in Table 2 when the genotypes of both parents are unknown, the range of the first parent exclusion probability (PE-1P) for a single locus is between 0.129 and 0.582. When the genotype of one parent is known, the range of the second parent exclusion probability (PE-2P) for a single locus is between 0.069 and 0.403. The cumulative exclusion probability for unknown parental genotypes (CEP1) is 0.9652999, while the cumulative exclusion probability for unknown parental genotypes (CEP2) is 0.9996999, as detailed in Table 2. The selected 16 microsatellite loci in the Yanqi horse population meet the requirement of a paternity exclusion probability of 0.99 for parentage testing and can be used to determine parentage. Furthermore, the selected microsatellite markers demonstrate their robustness in parentage identification, ensuring accurate recognition of genetic relationships within the population.

After eliminating two invalid samples, 115 Yanqi horse individuals were tested for paternity. Using the Simulation module to simulate 10,000 offspring, LOD results of 13.31 and 15.42 were obtained under relaxed (0.90) and strict (0.99) threshold conditions, respectively. The values 13.31 and 15.42 represent the LOD scores obtained during the simulation analysis, indicating high statistical significance for parent–offspring relationships under 90% and 95% confidence levels, respectively. The individual identification accuracy of the 16 microsatellite loci reached 100%. When only the maternal or paternal genotype is known, a few individuals could not be successfully identified using the Delta method. However, when the LOD method was employed to conduct simulations under three different conditions, all individuals were successfully identified. This demonstrates that when the genotypes of both parents are known, the identification accuracy is significantly higher compared to scenarios where only the maternal or paternal genotypes are known. Additionally, the accuracy of maternity identification is higher than that of paternity identification, which is related to the larger number of mares in the conservation group. Based on these results, the LOD method was utilized for subsequent paternity testing of individuals in the protected population. As shown in Table 3, among the 115 individuals who participated in the parent–child relationship identification, 52 offspring were ultimately determined to have a father–offspring relationship through microsatellite marker analysis, while 63 offspring were confirmed to have a mother–offspring relationship, achieving an accuracy of 90% in both cases. The confirmed parent–child relationships were further compared against paper pedigrees. Among the 115 individuals, 47 had missing parents in the original pedigree records, accounting for 40.2%, while 26 individuals had incorrect original pedigree records, representing 22.2%.

The LOD critical value in Cervus V3.0 was employed to conduct paternity testing on all individuals. The parentage testing results demonstrated higher confidence levels compared to previous studies using similar methods, with LOD scores surpassing the threshold values for both 90% and 95% confidence levels. A Supplementary Table (Table S3) is provided, containing all parentage testing results, enabling readers to assess the full range of confidence levels. Additionally, SPAGeDi V1.5 was utilized to calculate the kinship coefficients between parent–child pairs. The most likely parentage information for all 115 individuals in the Yanqi Horse population is presented in Table 4. In a population at Hardy–Weinberg equilibrium, the relatedness coefficient between individuals is 0.5, indicating a direct kinship relationship, such as that between parents and their offspring or between full siblings. A kinship coefficient of 0.25 suggests a half-sibling relationship. Based on the paternity test results from Cervus, the most probable parents of the offspring were identified. This information, combined with the relatedness coefficients of the three individuals, ultimately allowed for the determination of parent–child relationships. The parentage testing results exhibited higher confidence levels compared to previous studies using similar methods, with LOD scores exceeding the threshold values for 90% and 95% confidence levels. The Supplementary Table (Table S1) provides detailed results, including cases with both high and low confidence levels, offering a comprehensive overview of the findings.

### 3.3. Genetic Feature Analysis

Using Fstat software(v.2.9.4), genetic diversity and differentiation were estimated and tested through co-dominant genetic markers. Population genetic structure analysis based on microsatellite loci revealed significant genetic differentiation within the Yanqi horse population (Table 5). The F-statistics analysis indicated moderate levels of genetic differentiation, with an average FST value of 0.13, suggesting intermediate genetic divergence among subpopulations. The overall inbreeding coefficient (FIT) reached 0.47, reflecting moderate inbreeding at both individual and subpopulation levels. Notably, the average individual inbreeding coefficient (FIS) was 0.40, implying potential evolutionary processes such as inbreeding, genetic drift, or selection pressure within the population.

**Figure 2:**
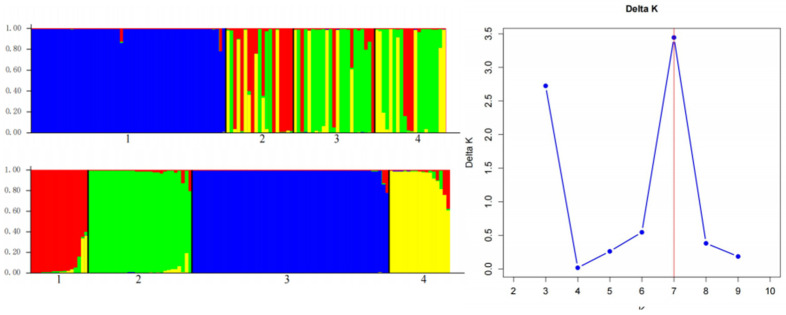
Population structure of the Yanqi horse. The clustering results illustrate the structural analysis of 117 Yanqi horses based on 16 microsatellite markers. Each horse’s genotype is represented by a vertical line, which is divided into K colors, where K denotes the number of clusters hypothesized in each structural analysis. Each bar corresponds to an individual horse, and the color on each vertical bar indicates the probability of that individual belonging to each cluster.

To further elucidate the population structure, we conducted Bayesian clustering analysis using Structure 2.3.4 software. The analysis was performed with K values ranging from 2 to 10, with 15 independent simulations for each K value. The optimal number of genetic clusters, determined by ΔK analysis using Structure Harvester v0.6.94, was identified as K = 4 (ΔK = 0.26), corresponding to the four sampling regions (Figure 2). The genetic structure analysis revealed a distinct geographic pattern of genetic differentiation. Individuals from Region A predominantly (85.3%) belonged to genetic cluster I, exhibiting unique allele frequency characteristics. Region B individuals were mainly (78.6%) assigned to cluster II, with approximately 15% showing admixture with Region C. Region C individuals were primarily (82.1%) distributed in cluster III, while about 12% displayed genetic admixture with Region B, indicating historical gene flow between these regions. Region D individuals exhibited the highest genetic distinctiveness, with 91.2% belonging to cluster IV.

Genetic distance analysis supported the observed geographic differentiation patterns. The average genetic distance within regions was 0.15, while inter-regional distances averaged 0.32. The maximum genetic distance (0.38) was observed between Regions A and D, consistent with their geographical separation. The relatively smaller genetic distance (0.25) between Regions B and C provided further evidence of gene flow between these areas. These findings demonstrate that the Yanqi horse population has developed unique genetic characteristics through long-term geographic isolation, forming relatively independent genetic units. The observed genetic structure, characterized by moderate differentiation and regional clustering, offers critical insights for conservation strategies. We recommend managing the four regional populations as separate conservation units while maintaining controlled gene flow between Regions B and C to preserve the population’s genetic diversity and evolutionary potential.

**Figure 3:**
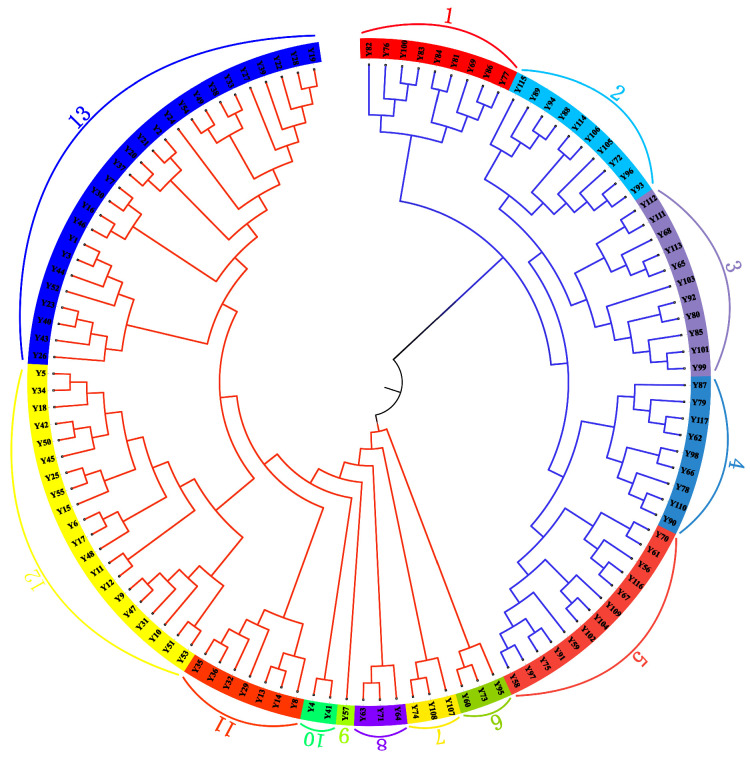
Phylogenetic tree based on 117 Yanqi horses in NJ. The number on each branch corresponds to each individual Yanqi horse.

The analysis based on Nei’s genetic distance and NJ phylogenetic tree revealed significant genetic differentiation within the Yanqi horse conservation population, which is primarily divided into two major clades see Figure 3. The first clade comprises family lines 1–5, with family lines 1 and 2 forming one cluster and family lines 3 and 4 forming another, both subsequently merging with family line 5 into a larger branch. This clustering pattern suggests a shared geographical origin for the founding individuals of these family lines. The second clade includes family lines 6–11, characterized by distinct sub-branching: three individuals from family line 6, a single individual from family line 7, and two individuals from family line 8 form independent sub-clusters, while family lines 9, 10, and 11 aggregate into a larger branch, with family line 11 being the most prominent. This phylogenetic structure likely reflects the breeding history of the population, where family lines 6–8 represent the early foundational stock, and family lines 9–11 may have originated through subsequent introduction and expansion of external genetic material. From a conservation genetics perspective, the high genetic similarity observed within the first clade underscores the need to minimize mating among family lines 1–5 to mitigate inbreeding risks. In contrast, the pronounced genetic differentiation between family lines 6–8 and 9–11 in the second clade highlights their potential as strategic hybridization pairs to enhance genetic diversity. These findings provide critical insights for refining breeding strategies in the Yanqi horse conservation program, ensuring the preservation of genetic diversity while effectively managing inbreeding depression.

## 4. Discussion

Genetic diversity is a core component of biodiversity and directly determines a species’ potential to adapt to environmental changes [24]. In the face of environmental pressures such as climate change, emerging diseases, or fluctuations in food resources, populations with higher genetic variation are more likely to contain individuals capable of adapting to new conditions, thereby ensuring long-term survival and evolution [25,26]. In this study, the genetic diversity of the Yanqi horse population was assessed using 16 microsatellite markers, revealing a total of 191 alleles, with an average allele number of 11.97, an observed heterozygosity (Ho) of 0.48, and a polymorphic information content (PIC) of 0.67. These results indicate that the Yanqi horse population exhibits moderate levels of genetic diversity at the selected microsatellite loci, although the population may have experienced inbreeding or genetic bottleneck effects. Compared to Mongolian and Kazakh horses, the Yanqi horse population shows relatively lower genetic diversity. Research by Jihye Yun (2022) on Mongolian horses, based on 14 microsatellite markers, reported an average allele number of 11.43, an observed heterozygosity of 0.76, and an average PIC of 0.74 [27]. For Kazakh horses, the average allele number was 13.467, the observed heterozygosity was 0.72, and the PIC ranged from 0.73 to 0.80 [28]. These differences may stem from the isolated habitat and unique breeding history of the Yanqi horse. Historically, Mongolian horses have significantly influenced the genetic composition of Yanqi horses [4], but long-term geographic isolation and artificial selection have led to the development of distinct genetic characteristics in Yanqi horses. Li Mengmeng’s (2022) analysis of the genetic structure of five local horse breeds in Xinjiang (including Yanqi horses) based on mtDNA provided important insights into the maternal genetic background of Yanqi horses [29]. However, systematic studies of Yanqi horses and other local horse breeds in Xinjiang using microsatellite markers or other nuclear gene markers are still lacking. The findings of this study not only fill a gap in the research on the genetic diversity of Yanqi horses but also provide crucial evidence for understanding their evolutionary history and adaptive potential. Additionally, they offer methodological references and data support for genetic diversity studies of other local horse breeds in Xinjiang, contributing to the systematic evaluation and conservation of genetic resources in the region.

Parentage identification is a critical component of animal genetic breeding, with its accuracy directly impacting the implementation of breeding programs. Currently, DNA-based molecular markers used for parentage identification include polymerase chain reaction (PCR) amplified fragment length polymorphism analysis, short tandem repeat (STR) analysis, single nucleotide polymorphism (SNP) analysis, gene chip technology, and next-generation sequencing [30,31,32,33]. Among these, STR analysis is the most widely used in animal parentage testing due to its high polymorphism, accuracy, and stability. In this study, parentage identification and molecular pedigree construction were performed for the Yanqi horse conservation population using 16 microsatellite markers. The average cumulative exclusion probability (CPE) exceeded 98%, and the reliability of parentage identification reached over 98%. Through Simulation analysis under conditions of 10,000 offspring, a lenient threshold (90%), and a strict threshold (95%), the accuracy rate of the 16 microsatellite markers reached 100%. Using the LOD method under three simulation conditions, parentage relationships were accurately determined for 115 individuals, validating the reliability of the parentage identification. Compared to parentage studies in other horse breeds, the results of this study demonstrate high reliability and practicality. For example, Song yang Shang (2022) developed a 19-plex PCR detection system through microsatellite primer redesign and multiplex PCR, achieving cumulative exclusion probabilities (CPE duo and CPE trio) of 0.994659935 and 0.999854032, respectively, suitable for individual identification and parentage testing in horses [34]. Wan yi Dang (2019) established a 13-plex STR typing system for donkey parentage testing, showing high exclusion probabilities (0.9889 and 0.9996), although the system cannot determine sex [35]. Oumaima (2024) compared the effectiveness of SNP and microsatellite markers in parentage testing for Moroccan horses, finding that SNP markers had a cumulative exclusion probability exceeding 99.99%, while microsatellite markers achieved exclusion probabilities of 99.8–99.9%, indicating the potential of SNPs in parentage testing but requiring further optimization of locus selection [14]. Although STR markers offer advantages such as high polymorphism, accuracy, and rapid detection in parentage testing, their limited loci and potential for gene mutations remain concerns. In the future, the integration of SNP, gene chip, or next-generation sequencing technologies may become the trend in parentage testing. These technologies not only improve resolution and accuracy but also provide more comprehensive genetic information, offering stronger technical support for animal breeding and genetic resource conservation [36,37].

Genetic differentiation is the process by which different populations of the same species gradually develop differences in genetic composition, with its extent reflecting the level of gene flow and evolutionary history among populations [38]. In this study, the genetic differentiation of the Yanqi horse population was systematically assessed using F-statistics and Bayesian clustering methods. F-statistics analysis revealed an average FST value of 0.13, indicating moderate genetic differentiation among populations; the average FIT and FIS values were 0.47 and 0.40, respectively, suggesting moderate inbreeding at both individual and subpopulation levels. Bayesian clustering analysis further supported these results, with the optimal number of genetic clusters identified as K = 4 (ΔK = 0.26), indicating that the Yanqi horse population may consist of four genetic subpopulations. Nei’s genetic distance-based clustering divided the Yanqi horses into 11 genetic groups, which exhibited high internal similarity and inter-group differentiation at multiple loci, consistent with the molecular pedigree results. The moderate genetic differentiation observed in the Yanqi horse population may be closely related to its geographical distribution and breeding history. Firstly, the Yanqi horses are primarily distributed in certain counties of the Bayingolin Mongol Autonomous Prefecture in Xinjiang, where their relatively concentrated distribution is partially isolated by natural barriers such as mountains and rivers, limiting gene flow across populations. Secondly, natural selection pressures in different ecological environments, such as basins and mountainous areas, may have led to genetic differentiation among populations, while ecological similarities maintained the stability of these differences. Additionally, breeding management practices have significantly influenced genetic differentiation: although the current breeding scale is large, historical fluctuations in population size have resulted in a small effective population size and the overuse of high-quality stallions combined with traditional breeding practices (e.g., local or within-subpopulation mating) have further exacerbated inbreeding. Compared to studies on other horse breeds, the Yanqi horses exhibit a higher level of genetic differentiation. For example, Agnieszka (2020) found that the average FST value for Polish Konik horses was only 0.0305, with negative FIS values at most loci, indicating low inbreeding levels in this population [39]. Bayesian clustering analysis showed that the number of genetic groups (K = 2) in Polish Konik horses did not match the actual number of pedigrees (6), suggesting that their genetic structure may be significantly influenced by human management practices. In contrast, the genetic differentiation pattern in Yanqi horses is more likely to reflect the combined effects of natural selection and geographic isolation. These findings provide important insights for the conservation of Yanqi horse genetic resources. It is essential to strengthen the protection of different genetic groups to prevent further loss of genetic diversity while prioritizing the mating of individuals with greater genetic distances in breeding management to reduce inbreeding risks. Future research could integrate genomic data to further elucidate the genetic basis of adaptive evolution in Yanqi horses, providing theoretical support for the development of scientific conservation strategies.

## 5. Conclusions

This study systematically analyzed the genetic diversity, parentage relationships, and genetic structure of the Yanqi horse conservation population using microsatellite markers. The results indicate that the Yanqi horse population exhibits moderate genetic diversity (average allele number: 11.969, observed heterozygosity: 0.481), with a parentage identification accuracy of 99%. Genetic differentiation analysis revealed moderate genetic divergence among populations (FST = 0.128) and moderate inbreeding levels (FIS = 0.396). Bayesian clustering analysis (K = 4) highlighted the impact of geographical isolation on genetic structure. The findings provide a scientific basis for the conservation and sustainable utilization of Yanqi horse genetic resources. It is recommended to strengthen conservation measures, optimize breeding strategies, and further investigate the genetic background using genomic technologies.

## Figures and Tables

**Table 1:** Genetic parameters of microsatellite loci.

Locis	Na	Ne	Ho	He	PIC	I
*HMS7*	12.00	8.02	0.54	0.86	0.78	0.60
*HTG10*	15.25	8.70	0.54	0.88	0.67	0.27
*ABS23*	16.75	11.08	0.73	0.90	0.85	0.01
*HMS3*	14.75	10.98	0.63	0.91	0.85	0.16
*AHT4*	10.25	5.97	0.32	0.79	0.62	0.73
*HMS2*	11.75	8.50	0.56	0.83	0.71	0.13
*TKY297*	11.75	8.57	0.46	0.82	0.65	0.59
*ABS17*	17.50	12.04	0.65	0.90	0.90	0.18
*AHT5*	14.25	10.88	0.52	0.82	0.71	0.08
*ABS2*	12.25	6.76	0.54	0.79	0.73	0.36
*HMS9*	7.75	4.26	0.29	0.73	0.65	0.84
*VHL20*	8.00	3.71	0.28	0.66	0.51	0.84
*HMS6*	8.00	4.60	0.36	0.73	0.51	0.55
*HMS18*	6.00	2.96	0.32	0.48	0.31	0.84
*TKY343*	15.00	5.31	0.64	0.80	0.69	0.39
*TKY337*	10.25	3.65	0.32	0.72	0.50	0.71
Mean	11.97	7.27	0.48	0.70	0.67	0.44

In this study, we examined several genetic parameters associated with the microsatellite marker Locis. The number of alleles (Na) was assessed alongside the effective number of alleles (Ne), observed heterozygosity (Ho), expected heterozygosity (He), polymorphic information content (PIC), and Shannon information index (I). Additionally, we provide the mean values for these metrics to summarize our findings.

**Table 2:** Exclusion probability of 16 microsatellite DNA polymorphic sites in the Yanqi horse population.

Locus	NE-1P	NE-2P
*HMS7*	0.25	0.14
*HTG10*	0.22	0.12
*ABS23*	0.19	0.11
*HMS3*	0.19	0.11
*AHT4*	0.30	0.18
*HMS2*	0.24	0.14
*TKY297*	0.27	0.16
*ASB17*	0.13	0.07
*AHT5*	0.22	0.12
*ABS2*	0.26	0.15
*HMS9*	0.38	0.24
*VHL20*	0.49	0.32
*HMS6*	0.39	0.24
*HMS18*	0.58	0.40
*TKY343*	0.32	0.19
*TKY337*	0.44	0.28
CEP1/CEP2	0.9652999	0.9996999

NE-1P refers to the probability of excluding the candidate parent based on the given genotypes of both the candidate parent and the offspring. NE-2P denotes the probability of excluding the candidate parent when considering the known parent genotype, the candidate parent genotype, and the offspring genotype. CEP1 represents the cumulative exclusion probability associated with unknown parent genotypes, while CEP2 indicates the cumulative exclusion probability for known parent genotypes.

**Table 3:** LOD—qualification simulation.

Identification Category	Confidence Level	Confidence (%)	LOD Threshold	Distribute	Distribution Rate
Maternity identification simulation.	Strict	95.00	4.08	4535	45%
	Relaxed	90.00	2.83	5034	50%
	Unassigned			4966	50%
	Total			10,000	100%
Paternity test simulation.	Strict	95.00	3.31	4758	48%
	Relaxed	90.00	1.86	5212	52%
	Unassigned			4788	48%
	Total			10,000	100%
Parentage Test Simulation.	Strict	95.00	15.42	1883	19%
	Relaxed	90.00	13.31	2261	23%
	Unassigned			7739	77%
	Total			10,000	100%

**Table 4:** Some individual paternity test results.

Offspring	Candidate Mother	LOD Threshold	Confidence	Candidate Father	LOD Threshold	Confidence
Y2	Y20	0.68	*	Y21	14.00	*
Y3	Y34	6.32	*	Y51	16.30	*
Y7	Y44	4.23	*	Y14	10.30	*
Y9	Y20	5.40	*	Y50	1.60	*
Y13	Y9	9.26	*	Y15	9.07	*
Y15	Y45	6.20	*	Y50	5.04	*
Y21	Y20	4.08	*	Y41	6.25	*
Y22	Y19	9.13	*	Y18	14.40	*
Y25	Y6	3.10	*	Y28	3.65	*
Y26	Y37	5.37	*	Y28	6.52	*
Y27	Y5	2.81	*	Y24	3.93	*
Y29	Y35	21.60	*	Y14	2.15	*
Y30	Y16	8.61	*	Y24	14.30	*
Y32	Y36	7.41	*	Y14	8.91	*
Y35	Y36	2.03	*	Y41	5.91	*
Y36	Y7	5.32	*	Y41	2.67	*
Y38	Y39	9.98	*	Y33	4.26	*
Y39	Y36	4.31	*	Y22	3.71	*
Y42	Y5	4.92	*	Y50	0.31	*
Y43	Y42	0.52	*	Y13	6.27	*
Y44	Y7	4.23	*	Y33	6.40	*
Y45	Y10	8.60	*	Y50	4.52	*
Y46	Y37	7.76	*	Y21	20.01	*
Y47	Y10	6.86	*	Y13	12.30	*
Y50	Y42	0.31	*	Y18	1.99	*
Y63	Y71	2.13	*	Y60	41.20	*
Y67	Y96	8.51	*	Y78	25.10	*

The asterisk (*) indicates results with a confidence level below the threshold (e.g., <95%).

**Table 5:** Population genetic parameters of microsatellite loci.

Locus	Ho	FST	FIT	FIS
*HMS7*	0.54	0.07	0.42	0.38
*HTG10*	0.54	0.06	0.41	0.38
*ABS23*	0.73	0.04	0.23	0.19
*HMS3*	0.63	0.04	0.34	0.31
*AHT4*	0.32	0.12	0.65	0.60
*HMS2*	0.56	0.10	0.39	0.32
*TKY297*	0.46	0.10	0.50	0.44
*ASB17*	0.65	0.07	0.32	0.27
*AHT5*	0.52	0.11	0.43	0.36
*ABS2*	0.54	0.13	0.40	0.31
*HMS9*	0.29	0.16	0.66	0.60
*VHL20*	0.28	0.22	0.67	0.58
*HMS6*	0.36	0.16	0.59	0.51
*HMS18*	0.32	0.36	0.58	0.34
*TKY343*	0.64	0.12	0.29	0.20
*TKY337*	0.32	0.17	0.63	0.55
Mean	0.48	0.13	0.47	0.40

## Data Availability

All data supporting the findings of this study are available within the article.

## Supplementary Materials

The following supporting information can be downloaded at: https://www.mdpi.com/article/10.3390/genes16030294/s1.

## References

[1] Hou W. (1997). Product Horse Breeding Science.

[2] Erdembayil H., Xu J., Yang Y. (2022). Ideas for the protection, development and utilization of genetic resources of Yanqi horses. China Livest. Poult. Breed. Ind..

[3] Li H. (2022). Research on the Appearance Characteristics and Biological Characteristics of Local Breed Horses in Xinjiang.

[4] Guan Y., Tserendorj, Niman (2014). Analysis of Yanqi horse germplasm characteristics and research on breeding directions. Herbiv. Livest..

[5] Guan Y., Cairen D., Niman (2014). Analysis and research on endurance performance of Yanqi horses. Herbiv. Livest..

[6] Rustam Y. (2023). Study on the Population Genetic Structure and Gait Trait Selection Signals of Yanqi Horses.

[7] Li Y. (2015). Survey and Analysis of Intestinal Nematodes of Yanqi Horses in Hejing. Grass-Feed. Livest..

[8] Nomindalai G., Li L., Liu Y., Bayin C., Hulcha (2023). Detection and sequence analysis of equine piroplasmosis etiology in some Yanqi horse farms in Xinjiang. China Anim. Quar..

[9] Tong P.P., Song X., Zhang A., Ren M., Zhang L., Fong L., Xie J. (2021). Serological investigation of equine viral arteritis in some areas of Xinjiang. Prog. Vet. Med..

[10] Cairen D., Guan Y., Hu X., Liu J., Meng J., Liu Y., Li M., Yao X., Niman (2014). Analysis and Research on Yanqi Horses Breed Characteristic and Breeding Direction. Grass-Feed. Livest..

[11] Rustanmu Y., Gao X., Li M., Qi A., Erdembayil (2024). Study on the correlation between contralateral gait and DMRT3 gene variation in Yanqi horses. China. J. Anim. Husb..

[12] Li H., Alifizhe U., Tang C., Geminguli M. (2021). Analysis of phenotypic differences in different types of Yanqi horses. Chin. Herbiv. Sci..

[13] Wang J., Yao X., Ren W., Wang C., Chu H., Luo P., Yan M., Mon J. (2020). Analysis of ACTN3 gene polymorphisms in Yanqi horses and their correlation with endurance race speed. Chin. J. Anim. Husb..

[14] Aminou O., Badaoui B., Machmoum M., Piro M. (2024). Evaluation of the effectiveness of single nucleotide polymorphisms compared to microsatellite markers for parentage verification in Moroccan horses. Anim. Genet..

[15] Flynn P., Morrin-O’Donnell R., Weld R., Gargan L.M., Carlsson J., Daly S., Suren H., Siddavatam P., Gujjula K.R. (2021). Comparative analysis of single nucleotide polymorphisms and microsatellite markers for parentage verification and discovery within the equine Thoroughbred breed. bioRxiv.

[16] Thelingwani R., Jonhera C.A., Masimirembwa C. (2024). Analysis of data and common mutations encountered during routine parentage testing in Zimbabwe. Sci. Rep..

[17] Damour G., Baumer K., Legardeur H., Hall D. (2023). Early noninvasive prenatal paternity testing by targeted fetal DNA analysis. Sci. Rep..

[18] Amos W., Flint J., Xu X. (2008). Heterozygosity increases microsatellite mutation rate, linking it to demographic history. BMC Genet..

[19] Smouse P.E., Peakall R. (1999). Spatial autocorrelation analysis of individual multiallele and multilocus genetic structure. Heredity.

[20] Weir B.S., Cockerham C.C. (1984). Estimating F-Statistics for the Analysis of Population Structure. Evolution.

[21] Falush D., Stephens M., Pritchard J.K. (2007). Inference of population structure using multilocus genotype data: Dominant markers and null alleles. Mol. Ecol. Notes.

[22] Hubisz M.J., Falush D., Stephens M., Pritchard J.K. (2009). Inferring weak population structure with the assistance of sample group information. Mol. Ecol. Resour..

[23] Jakobsson M., Rosenberg N.A. (2007). CLUMPP: A cluster matching and permutation program for dealing with label switching and multimodality in analysis of population structure. Bioinformatics.

[24] Sang Y., Long Z., Dan X., Feng J., Shi T., Jia C., Zhang X., Lai Q., Yang G., Zhang H. (2022). Genomic insights into local adaptation and future climate-induced vulnerability of a keystone forest tree in East Asia. Nat. Commun..

[25] Schierenbeck K.A. (2017). Population-level genetic variation and climate change in a biodiversity hotspot. Ann. Bot..

[26] King K.C., Lively C.M. (2012). Does genetic diversity limit disease spread in natural host populations?. Heredity.

[27] Yun J., Oyungerel B., Kong H.S. (2022). Genetic diversity and population structure of Mongolian regional horses with 14 microsatellite markers. Anim. Biosci..

[28] Ding L. (2017). Research on Phenotypic and Microsatellite Genetic Diversity of Kazakh Horses.

[29] Li M. (2022). Research on the Genetic Structure of Ili Horse Population.

[30] Zhang J., Liu K., Hao J. (2024). Fetal STR typing and paternity testing of first-trimester abortion tissue based on next-generation sequencing technology. China. J. Forensic Med..

[31] Guo J., Shang L., Tang Z. (2023). Construction and verification of a multi-source genetic marker detection system based on second-generation sequencing technology. Crim. Technol..

[32] Long J. (2021). Parentage analysis using genome-wide high-density SNP microarray. Gene.

[33] Škevin S., Tytgat O., Fauvart M., De Keyzer L., Deforce D., Van Nieuwerburgh F. (2023). STR genotyping by real-time PCR using QueSTR probes. Sens. Actuators B: Chem..

[34] Shang S., Jiang R., Luo R., Jia S., Irwin D.M., Wang Z., Zhang S. (2021). Development of a 19-plex short tandem repeat typing system for individual identification and parentage testing of horses (*Equus caballus*). Anim. Genet..

[35] Dang W., Shang S., Zhang X., Yu Y., Irwin D.M., Wang Z., Zhang S. (2019). A novel 13-plex STR typing system for individual identification and parentage testing of donkeys (*Equus asinus*). Equine Vet. J..

[36] Zhao Y., Wang K., Wang W.-L., Yin T.-T., Dong W.-Q., Xu C.-J. (2019). A high-throughput SNP discovery strategy for RNA-seq data. BMC Genom..

[37] Hu T., Chitnis N., Monos D., Dinh A. (2021). Next-generation sequencing technologies: An overview. Hum. Immunol..

[38] Xu L., Zhang K., Wang J. (2014). Exploring the Mechanisms of Differentiation, Dedifferentiation, Reprogramming and Transdifferentiation. PLoS ONE.

[39] Fornal A., Kowalska K., Zabek T., Piestrzynska-Kajtoch A., Musiał A.D., Ropka-Molik K. (2021). Genetic Variability and Population Structure of Polish Konik Horse Maternal Lines Based on Microsatellite Markers. Genes.

