# Supplementary material for: Identification of Genetic Relationships and Group Structure Analysis of Yanqi Horses
Source: Genes (Basel). 2025 Feb 27;16(3):294. doi: 10.3390/genes16030294 (PMC11941870; doi:10.3390/genes16030294)
Supplement: Supplementary file 1 [file genes-16-00294-s001.zip › LIst S1 translate.pdf]

Document of the Office of the National Committee on Livestock and Poultry Genetic Resources

No.1, 2021, of the Office of Livestock Resources Commission

Notice on the Publication of the List of National Livestock and Poultr Genetic Resources (2021 Edition)

To further enhance the relevance, standardization, and operability of the implementation of the "National Catalogue of Livestock and Poultry Genetic Resources," the National Committee on Livestock and Poultry Genetic Resources has organized the revision of the "National List of Livestock and Poultry Genetic Resources Varieties." This revision includes new livestock and poultry breeds, improved lines, and genetic resources that were approved or identified in

2020, as well as those previously overlooked. It also standardizes breed ranking and naming, corrects some content, and forms the "National List of Livestock and Poultry Genetic Resources Varieties (2021 Edition)," which includes 948 local breeds, developed breeds, introduced breeds, and improved lines. This list is now published and implemented.

The "National List of Livestock and Poultry Genetic Resources Varieties" announced on May 29,2020, is hereby repealed.

Office of the National Committee on Livestock and Poultry Genetic Resources

January 13,2021

horses

（1）Localvarieties

1.Abaga Black Horse

2.Oroqen horse

3.Mongolian horse

4.Tinny River horse

5.Jinjiang horse

6.Lichuanhorse

7.Baisehorse

8.Debao dwarf horse

9.Ganzi horse

10.Jianchang horse

11.Guizhou horse

12.Dali horse

13.Tengchong horse

14.Wenshan horse

15.Wumeng horse

16.Yongninghorse

17.Yunnan dwarf horse

18.Zhongdian horse

19.Tibetanhorse

20.Ningqianghorse

21.Chakou horse

22.Datong horse

23.Hequhorse

24.Chaidamu horse

25.Yushu horse

26.Barkol horse

27.Kazakh horse

28.Kyrgyzma horse

29.Yanqihorse

(3) Introduction of varieties

1 1.Sanhe Horse

2. Jinzhou Horse

3.Tieling Draft Horse

4.Jilin Horse

5.Guanzhong Horse

6.Bohai Horse

7.Shandan Horse

8.Yiwu Horse

9.Xilin Gol Horse

10.Horqin Horse

11.Zhangbei Horse

12.New Lijiang Horse

13.Ili Horse
