# Supplementary material for: Identification of Genetic Relationships and Group Structure Analysis of Yanqi Horses
Source: Genes (Basel). 2025 Feb 27;16(3):294. doi: 10.3390/genes16030294 (PMC11941870; doi:10.3390/genes16030294)
Supplement: Supplementary file 1 [file genes-16-00294-s001.zip › LIst S1.pdf]

国家畜禽遗传资源委员会办公室文件

Document of the Office of the National Committee on Livestock and Poultry Genetic Resources

畜资委办〔2021〕1 号

No.1, 2021, of the Office of Livestock Resources Commission

关于公布《国家畜禽遗传资源品种名录（2021 年版）》的通知

Notice on the Publication of the List of National Livestock and Poultry Genetic Resources (2021 Edition)

为进一步增强《国家畜禽遗传资源目录》贯彻实施的针对性、规范性和可操作性，国家畜禽遗传资源委员会组织开展了《国家畜禽遗传资源品种名录》修订工作，增加了 2020 年审定、鉴定通过的畜禽新品种、配套系和遗传资源，以及遗漏的畜禽品种、配套系和遗传资源，规范了品种排序、品种命名，对部分内容进行了勘误，形成《国家畜禽遗传资源品种名录(2021 年版)》，收录畜禽地方品种、培育品种、引入品种及配套系 948 个。现予以公布并实施。2020 年 5 月 29 日公布的《国家畜禽遗传资源品种名录》同时废止。

国家畜禽遗传资源委员会办公室

2021 年 1 月 13 日

四、马

（一）地方品种

1.阿巴嘎黑马
2.鄂伦春马
3.蒙古马
4.锡尼河马
5.晋江马
6.利川马
7.百色马
8.德保矮马
9.甘孜马
10.建昌马
11.贵州马
12.大理马
13.腾冲马
14.文山马
15.乌蒙马
16.永宁马
17.云南矮马
18.中甸马
19.西藏马
20.宁强马
21.岔口驿马
22.大通马
23.河曲马
24.柴达木马
25.玉树马
26.巴里坤马
27.哈萨克马
28.柯尔克孜马
29.焉耆马

（二）培育品种

1.三河马
2.金州马
3.铁岭挽马
4.吉林马
5.关中马
6.渤海马
7.山丹马
8.伊吾马
9.锡林郭勒马
10.科尔沁马
11.张北马
12.新丽江马
13.伊犁马

（三）引入品种

1.纯血马
2.阿哈-捷金马
3.顿河马
4.卡巴金马
5.奥尔洛夫快步马
6.阿尔登马
7.阿拉伯马
8.新吉尔吉斯马
9.温血马（荷斯坦马、荷兰温血马、丹麦温血马、汉诺威马、奥登堡马、塞拉-法兰西马）
10.设特兰马
11.夸特马
12.法国速步马
13.弗里斯兰马
