# Supplementary figures and images for: Identification of Genetic Relationships and Group Structure Analysis of Yanqi Horses
Source: Genes (Basel). 2025 Feb 27;16(3):294. doi: 10.3390/genes16030294 (PMC11941870; doi:10.3390/genes16030294)

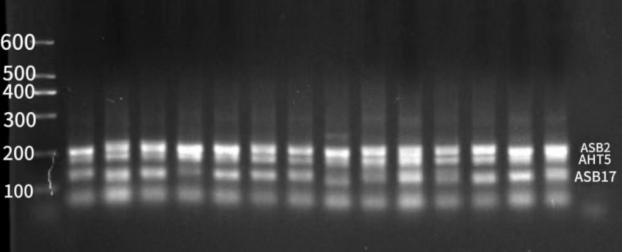

Supplement: Supplementary file 1 [file genes-16-00294-s001.zip › 1 ASB2 AHT5 ASB17 pcr product.jpg]

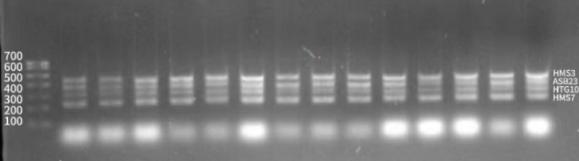

Supplement: Supplementary file 1 [file genes-16-00294-s001.zip › 2 HMS7、HTG10、ASB23、HMS3 pcr product.jpg]

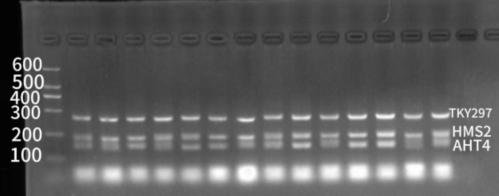

Supplement: Supplementary file 1 [file genes-16-00294-s001.zip › 3 TKY297 HMS2 AHT4pcr product.jpg]

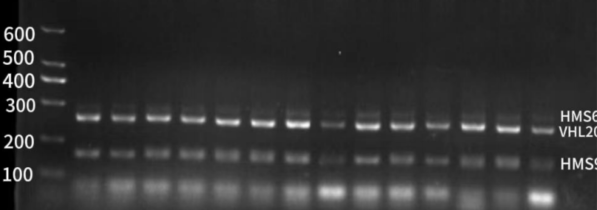

Supplement: Supplementary file 1 [file genes-16-00294-s001.zip › 4 HMS6 VHL20 HMS9pcr product.png]

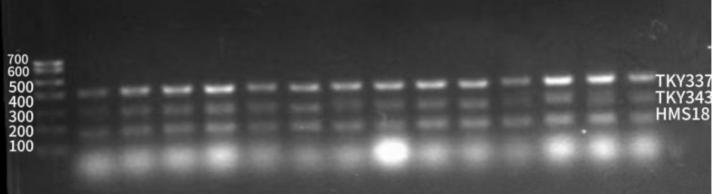

Supplement: Supplementary file 1 [file genes-16-00294-s001.zip › 5 TKY337 TKY343 HMS18 pcr product.jpg]
